# Comparative Analysis of Circular RNAs Expression and Function between Aortic and Intracranial Aneurysms

**DOI:** 10.2174/0113894501319306240819052840

**Published:** 2024-08-30

**Authors:** Ilgiz Gareev, Ozal Beylerli, Aamir Ahmad, Tatiana Ilyasova, Huaizhang Shi, Vladimir Chekhonin

**Affiliations:** 1 Central Research Laboratory, Bashkir State Medical University, Ufa, 450008, Russia;; 2 Educational and Scientific Institute of Neurosurgery, Peoples' Friendship University of Russia (RUDN University), Moscow, Russia;; 3 Translational Research Institute, Academic Health System, Hamad Medical Corporation, Doha, Qatar;; 4 Department of Neurosurgery, The First Affiliated Hospital of Harbin Medical University, Harbin, 1500, China;; 5 Pirogov Russian National Research Medical University of the Ministry of Healthcare of Russian Federation, Moscow, Russian Federation;; 6 Serbsky Federal Medical Research Centre of Psychiatry and Narcology of the Ministry of Healthcare of Russian Federation, Moscow, Russian Federation;; 7 The National Medical Research Center for Endocrinology, Moscow, Russian Federation

**Keywords:** Circular RNA, thoracic aortic aneurysm, abdominal aortic, intracranial aneurysm, subarachnoid hemorrhage, dissection, expression, therapeutic targets, biomarker, analysis

## Abstract

An aneurysm is an abnormal enlargement or bulging of the wall of a blood vessel. Most often, aneurysms occur in large blood vessels - the aorta (Thoracic Aortic Aneurysm (TAA) and Abdominal Aortic Aneurysm (AAA)) and brain vessels (Intracranial Aneurysm (IA)). Despite the presence of significant differences in the pathogenesis of the development and progression of IA and TAA/AAA, there are also similarities. For instance, both have been shown to be strongly influenced by shear stress, inflammatory processes, and enzymatic destruction of the elastic lamellae and extracellular matrix (ECM) proteins of the vascular wall. Moreover, although IA and TAA are predominantly considered arteriopathies with different pathological mechanisms, they share risk factors with AAA, such as hypertension and smoking. However, there is a need for a more in-depth study of the key elements that may influence the formation and progression of a particular aneurysm to find ways of therapeutic intervention or search for a diagnostic tool. Today, it is known that the disruption of gene expression is one of the main mechanisms that contribute to the development of aneurysms. At the same time, growing evidence suggests that aberrant epigenetic regulation of gene function is strongly related to the genesis of aneurysms. Although much has been studied of the known protein-coding genes, circular RNAs (circRNAs), a relatively new and rapidly evolving large family of transcripts, have recently received much scientific attention. CircRNAs regulate gene expression through the sponging of microRNAs (miRNAs) and can also be used as therapeutic targets and biomarkers. Increasing evidence has implicated circRNAs in the pathogenesis of multiple cardiovascular diseases, including the development of aneurysms. However, the mechanism of dysregulation of certain circRNAs in a particular aneurysm remains to be studied. The discovery of circRNAs has recently advanced our understanding of the latest mode of miRNAs/target genes regulation in the development and progression of IA and TAA/AAA. The aim of this study is to compare the expression profiles of circRNAs to search for similar or different effects of certain circRNAs on the formation and progression of IA and TAA/AAA.

## 1 INTRODUCTION

An aneurysm is a pathological expansion of an arterial vessel by more than 50% compared to a constant diameter. Segmental dilatation of the artery occurs because of weakening (damage) of the vascular wall. Most often, aneurysms form in the aorta: Abdominal Aortic Aneurysms (AAAs) has a prevalence that ranges between 1.3% and 18% with a prevalence of 0.16% for Thoracic Aortic Aneurysms (TAAs). Aortic Aneurysm Dissection (AAD) is known to be that dangerous condition. ADD has a high mortality rate because separation of the media and adventitia caused by intimal rupture of the vascular wall can lead to excessive internal bleeding within a couple of hours. At present, it has not been clinically proven that conservative treatment can effectively prevent disease progression [1, 2]. Another type of aneurysms, Intracranial Aneurysms (IAs) are also found in the arterial vessels that supply the brain; however, they are often diagnosed after Subarachnoid Hemorrhage (SAH) resulting from a ruptured aneurysm. SAH has high mortality and morbidity, with an incidence of 6-8/100 000. One-year survival costs after SAH are 3 times higher than elective surgical or endovascular treatment for patients with an unruptured aneurysm [3, 4]. Therefore, understanding the biological mechanisms of arterial aneurysm formation and progression can potentially help optimize early diagnosis, prognosis (probability of IAs rupture and AAD), and treatment of patients.

Recent studies have identified a new class of non-coding RNAs (ncRNAs) called circular RNAs (circRNAs), which are derived from the fusion of exons or introns or both introns into covalently closed rings [5]. Although the functional significance and mechanism of action of circRNAs are still under active investigation, they have been shown to regulate the expression of microRNAs (miRNA sponge) and target genes, as well as the sequestration of RNA-binding proteins [5]. Aberrant circRNAs expression is thought to cause a number of disorders, including aneurysms formation and progression, accompanied by key mechanisms such as vascular extracellular matrix (ECM) degradation, endothelial dysfunction, altered Vascular Smooth Muscle Cells (VSMCs) phenotype and apoptosis, and chronic inflammatory processes [6, 7]. Moreover, it is known that patients with IAs, TAAs, and AAAs usually suffer from many other diseases, such as hypertension and atherosclerosis, and have a long history of smoking [2, 4]. These factors have their own distinctive circRNA expression profiles [6, 7]. SincecircRNAs have unique biological functions and molecular characteristics, they have potential applications as novel nucleic acid-based therapeutic agents. One possible application is the use of circRNAs as stable molecules for RNA interference therapy [8]. Based on the stability and biological functions of circRNAs, various applications of these RNAs are being explored, including miRNAs and protein decoys, stable antisense RNAs, enhancers/ inhibitors of innate immune responses, and templates for protein translation [9, 10]. These various circRNAs-based treatments may also be applicable in the early stages of aneurysm development when the processes are still reversible.

These unusual features also make circRNAs potentially suitable molecular biomarkers, especially for aneurysms and their complications. Unlike linear RNAs, circRNAs are covalently closed single-stranded circular transcripts without a 5' cap or 3' poly(A) tail. This special structure makes them stable and conserved, and they are dynamically expressed in specific tissues through a unique process [11, 12]. Since circRNAs have the advantage of cell, tissue, or developmental stage-specific expression, and IAs, TAAs, or AAAs are already known to have different developmental mechanisms, circRNAs can also be used to classify and identify a particular aneurysm. The unique characteristics and biological functions of circRNAs indicate that they have the potential to become promising biomarkers for aneurysm prediction, diagnosis, and prognosis, as well as therapeutic targets. In this study, we aim to analyze the role of circRNAs as critical regulators of IA and TAAs/AAAs pathogenesis and as biomarkers, as well as information about the similarities or differences in changes in the expression profile of certain circRNAs. To the best of our knowledge, this is the first study to achieve this purpose.

## 2 MECHANISM DEVELOPMENT AND PROGRESSION OF ANEURYSMS

In general, aneurysms can form from the arterial and venous walls of vessels, but they tend to develop predominantly from their arteries. As mentioned, the two most common types of aneurysms are TAAs/AAAs and IAs. In addition to different locations, most TAAs and AAAs are fusiform in shape, whereas IAs are saccular in shape. While patients with TAAs and AAAs are studied and treated primarily by vascular surgeons, IAs patients are primarily treated by neurosurgeons and neurologists [1, 3]. Since the danger of both TAAs/AAAs and IAs lies in the dissection/rupture of the aneurysm, the question arises whether these types of aneurysms are inherently different or whether they have similar etiology, pathogenesis, and biomechanics.

The desire to identify and explain the underlying mechanisms of aneurysm formation and progression is an ongoing and widely debated issue in current research. While some aneurysms remain stable, others grow as the vessel wall thins and eventually ruptures. Aneurysm rupture occurs in terms of the mechanical effect of blood flow when the shear stress acting on the wall locally exceeds the strength of the wall to damage it [13]. The pathogenesis of IAs involves persistent pathological remodeling of cerebral vessels with degradation of the extracellular matrix through Matrix Metalloproteinases (MMPs) and apoptosis with concomitant inflammation of the vascular wall [14]. In addition, there is a strong relationship between wall shear stress, endothelial dysfunction, and the subsequent inflammatory response [14]. High concentrations of the enzyme cathepsin (cathepsin B, cathepsin K, and cathepsin S) and decreased concentrations of their most common inhibitor, cystatin C, have been shown to be involved in the pathogenesis of IAs [15]. It is assumed that oxidative stress and the influence of Nitric Oxide (NO) also contribute to the pathogenesis of IA through degradation of the ECM and stimulation of the inflammatory process, changes in blood flow hemodynamics, increased phenotypic modulation of VSMCs and, ultimately, their death [14]. Available evidence indicates that oxidative stress in the aortic wall is closely related to the pathogenesis of TAAs/AAAs. Oxidative stress promotes the recruitment of immune cells into the vasculature by modulating inflammatory cytokines. In addition, Reactive Oxygen Species (ROS) alter the balance between damage and repair of the aortic wall, increasing MMPs activity [16]. Many members of the cysteine, cathepsin, and MMPs subfamilies are molecules with potent enzymatic properties that mediate ECM degradation, leading to acute AAD and TAAs/AAAs rupture [17]. At the same time, the molecules responsible for the balance of maintaining the activity of these enzymes, namely Tissue Inhibitors of Metalloproteinases (TIMPs), which play a key role in preventing ECM degradation due to their ability to inhibit MMPs, have low expression levels [17].

The discovery of circRNAs has recently advanced our understanding of new mechanisms in the development and progression of aneurysms. However, more research is needed to utilize these molecules in diagnostics or therapy fully. In particular, we need to discover the full spectrum of miRNAs and mRNAs target genes that may be targeted by a given circRNAs in the context of the pathogenesis of a given aneurysm, how these molecules functionally interact, and the extent to which each contributes to the observed circRNAs functionality. We will try to describe this issue in more detail below.

## 3 CIRCULAR RNA AND RISK FACTORS

The etiology of TAAs/AAAs and IAs are still controversial, but risk factors for IAs include congenital or inherited defects that weaken the arterial wall, such as hypertension and atherosclerosis, and female gender and smoking are risk factors for aneurysm growth and rupture [18]. Risk factors for TAAs and AAAs may include atherosclerosis of the aortic wall, although this remains controversial, and smoking appears to have a direct effect on the risk of developing TAAs and AAAs independent of atherosclerosis. In addition, collagen deficiency has been described in both TAAs/AAAs and IAs. To date, there is some information about the role of circRNAs in the regulation of these risk factors [19, 20].

### 3.1 Hypertension

The state of the endothelium is the main factor determining normal vascular function and its changes in hypertension. Endothelial dysfunction is believed to be one of the earliest reversible stages of vascular remodeling. It has been proven that endothelial function deteriorates significantly already in the early stages of hypertension development [21]. In previous studies, accumulative evidence has documented that circRNAs are critically involved in endothelial homeostasis [22, 23]. Overall, existing studies expand our current understanding of the mechanisms of action of circRNAs in the development of hypertension. However, studies on the effect of circRNAs on hypertensive endothelial dysfunction have not been comprehensively studied. Most importantly, whether circRNAs can be recognized as critical regulators in hypertension still requires a lot of research. Research is needed to understand the underlying mechanisms of action of circRNAs in endothelial dysfunction associated with hypertension, which will provide unique opportunities to understand the role of circRNAs in the mechanisms of aneurysm formation and progression.

In attempts to establish the vector of connection between endothelial dysfunction and hypertension, it is necessary not to forget about an important component directly involved in the formation of tone and blood pressure control, such as VSMCs [24, 25]. In particular, the change in the contractile phenotype of VSMCs to the synthetic phenotype leads to excessive proliferation and migration of these cells, as well as degradation of the ECM, thereby acting in concert to induce vascular remodeling in hypertension [24, 25]. Considering these results, targeting dysfunctional VSMCs may have significant implications for the treatment of hypertension and associated vascular pathologies such as aneurysms. Recent studies have also shown that circRNAs can regulate the proliferation and migration of VSMCs during the development of hypertension [26-28]. In contrast to miRNAs and long non-coding RNAs (lncRNAs), only a subset of circRNAs in VSMCs have been tested in the context of hypertension, and only a few VSMC-associated circRNAs have demonstrated their regulatory roles [29]. For instance, Gong *et al.* demonstrated that circEsyt2 can play the role of a “molecular switch” by binding and influencing the nuclear translocation of Poly(rC)-Binding Protein 1 (PCBP1), subsequently regulating p53 variable splicing and p53β production and thereby directly influencing VSMCs phenotypic switching and vascular structure [30]. Other circRNA, such as circWDR77, was found to regulate cellular phenotypes, such as VSMCs proliferation and migration, by acting as miRNA sponges [31]. Chen *et al.* performed circRNAs microarray analysis to screen the circRNAs expression profiles in high glucose-induced VSMCs *in vitro* and identified the regulatory pathway of circWDR77/miR-124/fibroblast growth factor 2 (FGF2) in VSMCs proliferation and migration. The results provided new insights that helped expand the current understanding of how circRNAs function in the pathogenesis of hypertension. However, the role and mechanisms of VSMCs-derived circRNAs in hypertension remain largely unexplored. As a consequence of all this, there is an urgent need to elucidate the pathological mechanisms of cirсRNAs in hypertension, thereby advancing the pathological mechanism in aneurysms. The expression profiles, target genes, and proposed functional roles of the most abundant circRNAs shown to be associated with hypertension are demonstrate in Figs. (**1** and **2**) [32-36].

### 3.2 Atherosclerosis

It has already been proven that atherosclerosis correlates with the occurrence of arterial aneurysms [37]. However, it is still unknown whether this is a cause-and-effect relationship or whether they have common risk factors. It is a fact that most studies agree that atherosclerosis is primarily correlated with AAAs rather than TAAs and IAs [38]. There is a common genetic basis for both AAAs and atherosclerosis, as well as common risk factors, including hypertension, obesity, smoking, High-Density Lipoprotein (HDL), family history, and thrombosis [39]. Endothelial Cells (ECs) and VSMCs, the most important components of cerebral vessels and aorta, play an important role in the occurrence and development of atherosclerosis [37]. As mentioned earlier, there are various circRNAs that can influence the proliferation and migration of VSMCs and ECs. Perhaps these circRNAs can be studied for aneurysms [34, 35]. Additionally, immune cells such as macrophages play a central role in the initiation of atherosclerosis. For instance, the research conducted by Chen *et al.* indicated that circ-BANP is linked to apoptosis and inflammation [40]. At the same time, it enhances cell viability and is associated with increased migration, invasion, and tube formation in ECs. In parallel, circHIPK3, through its interaction with miR-106a-5p, demonstrates opposing effects on VSMCs by inhibiting their proliferation and migration while promoting calcification. These seemingly contradictory mechanisms pose a challenge in determining whether they collectively contribute to the progression or attenuation of atherosclerosis. This complexity highlights the limitations of relying solely on *in vitro* analyses to draw definitive conclusions. Moreover, endothelial cell dysfunction, which is evident in the early stages of atherosclerosis, is closely linked to chronic inflammatory changes within the arterial walls. These inflammatory processes are crucial in the development and progression of atherosclerosis, further complicating the interpretation of the effects of circ-BANP and circHIPK3. Additionally, the potential inaccuracies and inconsistencies observed in experimental results raise concerns about the replicability and overall reliability of these study designs. Thus, while circ-BANP and circHIPK3 are significant players in cellular processes related to atherosclerosis, their precise roles in the disease's progression remain ambiguous. This ambiguity underscores the limitations of *in vitro* studies in fully capturing the complex dynamics of atherosclerosis. It also emphasizes the need for further validation through rigorous and reproducible research methodologies. To better comprehend these molecular mechanisms and their impact on atherosclerosis, more comprehensive studies, including *in vivo* models, are essential. Such studies would provide a clearer understanding of how these circRNAs influence the disease and help develop potential therapeutic strategies. Furthermore, addressing the potential issues with experimental accuracy and replicability is crucial to advance our knowledge in this area. This approach will ensure that the findings are robust and applicable to clinical settings, ultimately leading to more effective treatments for atherosclerosis. Focus on Figs. (**1** and **2**), which summarizes the latest research progress on the most abundant circRNAs, focusing on the molecular mechanisms of circRNAs in atherosclerosis [34, 35, 41-44].

### 3.3 Shear Stress and Smoking

**Fig. (1):**
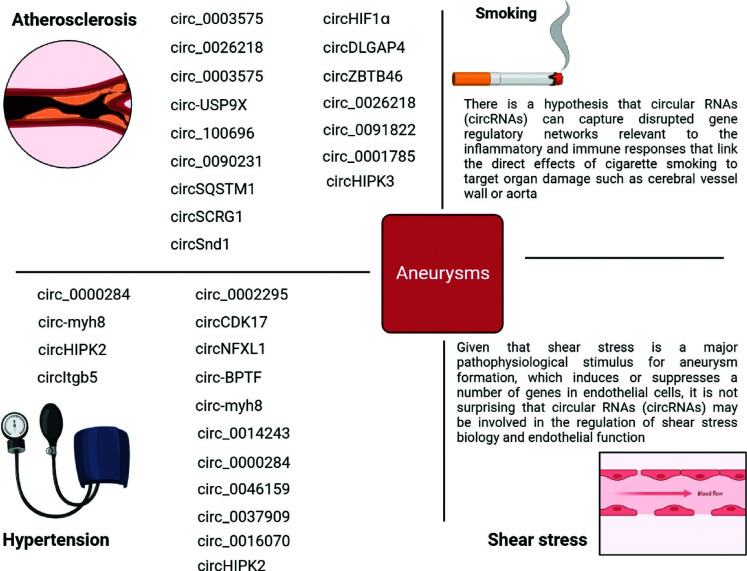
Main modifiable risk factors for aneurysms and circular RNAs (circRNAs). The above circRNAs can potentially be considered as new players in the formation of aneurysms through the molecular mechanisms of regulation of the pathogenesis of hypertension and atherosclerosis, as well as changes in their expression level under the influence of smoking and shear stress.

**Fig. (2):**
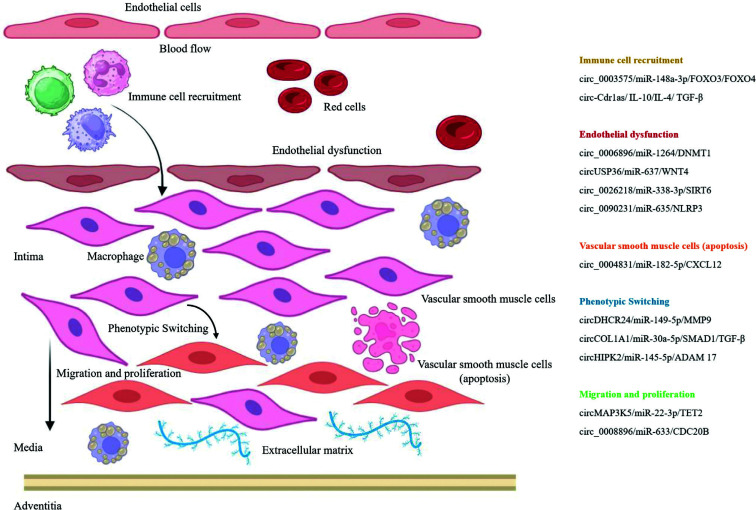
Circular RNAs (circRNAs) regulate vascular wall remodeling during hypertension and atherosclerosis processes. Considerable amounts of circRNAs, such as circ_0003575, circ-Cdr1as, circ_0006896, circUSP36, circ_0026218, circ_0090231, circ_0004831, circDHCR24, circCOL1A1, circHIPK2, circMAP3K5, and circ_0008896 through modulation of direct targets or in the presence of external stimuli (as immune cell recruitment) can promote the vascular wall remodeling.

The human circulatory system is quite complex, and many factors influence its functioning. Malfunctions in the circulatory system have significant negative consequences. Particularly vulnerable are the areas of narrowing, dilation, and branching of blood vessels, where the occurrence of an aneurysm or stenosis leads to impaired blood circulation [45]. Among other things, in certain situations, the aneurysm can enlarge until it ruptures, which can lead to the most unfavorable consequences. The walls of the cerebral arteries have structural differences from the extracranial (peripheral) arteries, consisting of a more poorly defined adventitia and a lower content of elastic fibers [46]. In addition, the cerebral arteries are immersed in the cerebrospinal fluid of the subarachnoid space to a greater extent than in the connective tissue. These structural features are thought to make cerebral arteries vulnerable to aneurysm formation [47]. The homeostasis of the cerebral artery wall can be influenced by risk factors for the development of IAs, such as smoking, which leads to impaired blood flow [48]. In response to the influence of this factor, structural changes occurring in the wall of the cerebral arteries lead to disturbances in the internal elastic plate in the area of the artery bifurcation. Aberrant blood flow leads to mechanical overload, wall stretch, and shear, which leads to recovery and degradation of the ECM due to apoptosis and/or modulation of VSMCs, as well as ECs dysfunction and macrophage influx. Sustained hemodynamic shear stress and smoking lead to ECM degradation, ECs dysfunction, and apoptosis or phenotypic modulation of VSMCs toward dedifferentiated, pro-inflammatory VSMCs [49]. Once molecular mechanisms no longer compensate for mechanical overload of the vessel wall and myointimal damage, cellular and humoral inflammatory responses become the main mechanisms of aneurysm formation. These processes, associated with the release of inflammatory cytokines such as tumor necrosis factor-alpha (TNF-α), interleukin 1β (IL-1β), and MMPs, promote the influx of macrophages and the continuous degradation of ECM [50, 51]. Wall shear stress and smoking may also promote cellular inflammatory responses during aneurysm formation. High wall shear stress and corresponding disruption of blood flow promote aneurysm formation and growth by remodeling (thinning) the vessel wall. In this case, small aneurysms develop. At the same time, low wall shear stress leads to destructive wall remodeling mediated by inflammation, the development of recirculation of intra-aneurysmal blood flow, which leads to the formation of relatively large, thick-walled arteriosclerotic aneurysms [7, 51, 52]. As already mentioned, circRNAs perform regulatory functions in the endothelium [34, 35]. However, the involvement of circRNAs transcripts in the regulation of shear stress sensing in ECs and changes in the expression profile during smoking remains unexplored and has not been investigated in terms of correlation with aneurysm formation. We have no doubt that there are circRNAs induced by shear stress and smoking. However, experimental research in this direction is necessary (Figs. **1** and **2**) [53-55].

## 4 CircRNAs IN ANEURYSMS

As already mentioned, IAs share common pathological features with TAAs and AAAs [18-20]. However, unlike the vast majority of cases of IAs or TAAs, cases of AAAs are more often associated with specific genetic factors and may clinically manifest as part of syndromic connective tissue diseases (*e.g.*, Marfan syndrome) [56]. To date, a relatively small number of circRNAs with proven biological significance for IAs, TAAs, and AAAs have been studied. Several studies have confirmed that circRNAs play a role in the formation and progression of IAs, TAAs, and AAAs (Tables **1** and **2**) [57-97].

Cytoskeleton-associated protein 4; ATM, Serine/threonine kinase; α-SMA, Smooth muscle cell α-actin; GSK3β, Glycogen synthase kinase-3 beta; LYPD3, LY6/PLAUR Domain containing 3; GRIA4, Glutamate receptor 4; SOD2, Superoxide dismutase 2; CCR7, Chemokine (C-C motif) receptor 7; PGRMC1, Progesterone receptor membrane component 1; CNN1, Calponin 1; IL-6, Interleukin 6; CCL2, C-C motif ligand 2; ERK, Extracellular-signal-regulated kinase; NF-κB, Nuclear factor kappa-light-chain-enhancer of activated B cells; RPS27A, 40S ribosomal protein S27a; ADAM10, Disintegrin and metalloproteinase domain-containing protein 10; CKAP4, Cytoskeleton-associated protein 4; BASP1, Brain acid soluble protein 1; COL4A1, Collagen, type IV, alpha 1; SM22α, Smooth muscle 22α; ZFP36, Zinc finger protein 36 homolog; Bcl-2, B-cell lymphoma 2; MMP2, Matrix metalloproteinase 2; MMP9, Matrix metalloproteinase 9; KLF4, Krüppel-like factor 4; TGFβR1, Transforming growth factor β receptor type 1.

It is well known that circRNAs play fundamental roles in vascular integrity and vascular function. Modern understanding of the role of circRNAs in the pathogenesis of aneurysms is largely based on studies of aneurysmal tissue of cerebral and aortic vessels obtained during interventional surgery for IAs and during open operations of TAAs and AAAs. Sincethese interventions are usually carried out in the later stages of the disease, minimal information is available to people about the initial role of the early stages of the disease. Questions remain as to whether the observed late changes in circRNA expression levels are a cause or a consequence of the disease.

**Fig. (3):**
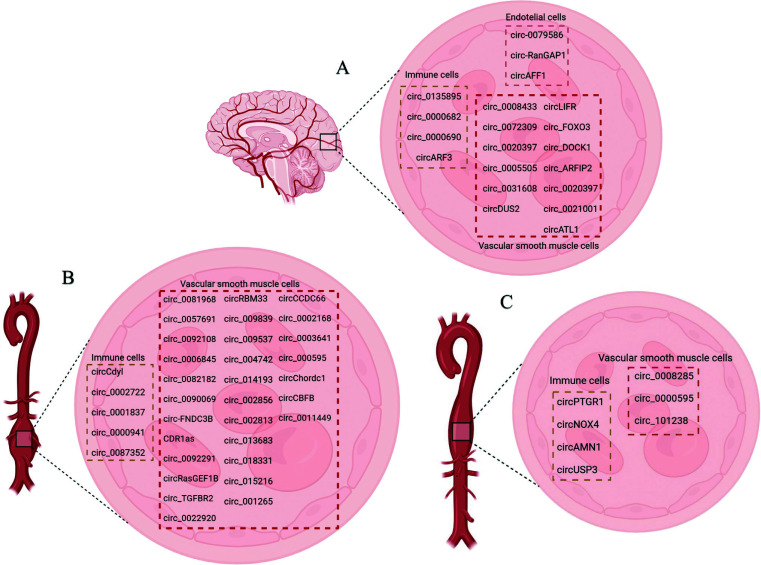
Examples of circular RNAs (circRNAs) which involved in thoracic aortic aneurysm (TAA), abdominal aortic aneurysm (AAA) and intracranial aneurysm (IA) (A-C). Several circRNAs involved in vascular endothelium dysfunction, in vascular smooth muscle cells (VSMCs) switching and inflammation in the vascular wall.

**Fig. (4):**
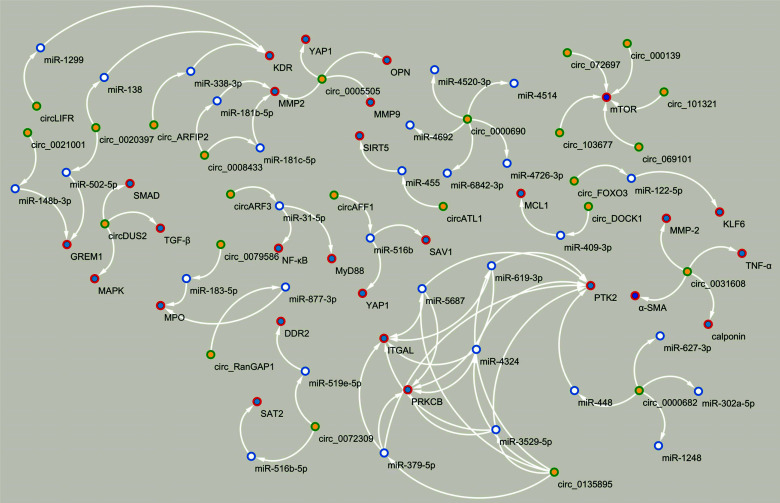
Circular RNAs (circRNAs) mediate the pathogenesis of intracranial aneurysm (IA) and subarachnoid hemorrhage (SAH) mainly through the following two major mechanisms such as circRNA-microRNA (miRNA)-messenger RNA (mRNA) axis and interaction with proteins. Note: This figure was made using Cytoscape (version 3.4.0).

**Fig. (5):**
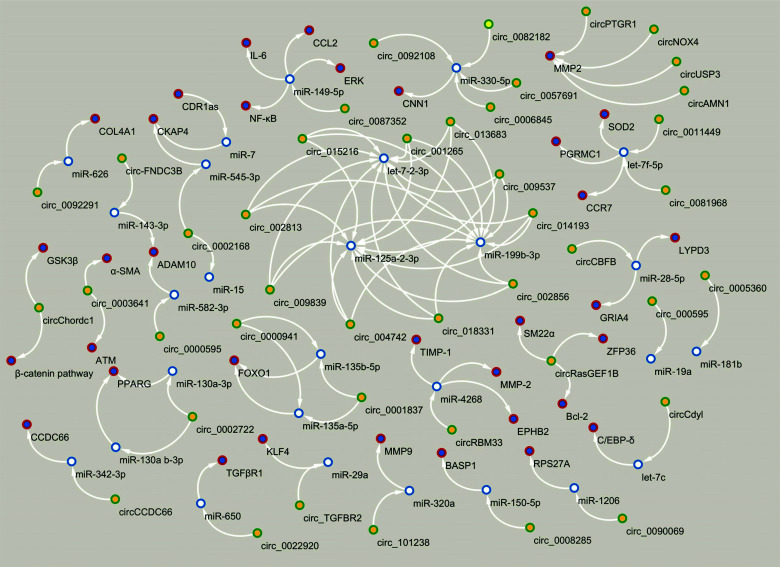
Circular RNAs (circRNAs) mediate the pathogenesis of thoracic aortic aneurysm (TAA), abdominal aortic aneurysm (AAA), and acute aortic dissection (AAD) mainly through the following two major mechanisms such as circRNA-microRNA (miRNA)-messenger RNA (mRNA) axis and interaction with proteins. Note: This figure was made using Cytoscape (version 3.4.0).

The process of IAs, TAAs, and AAAs formation involves several subtypes of vascular cells such as ECs, VSMCs, immune cells, and adventitial cells, where circRNAs are aberrantly expressed in these vascular wall cells during aneurysm development (Fig. **3A**-**C**) [57-97]. The mechanism of endothelial dysfunction, phenotypic reprogramming of VSMCs and their apoptosis, chronic inflammation, and oxidative stress (see “Mechanism development and progression of aneurysms”) is regulated under the control of certain circRNAs through the circRNAs-miRNAs-mRNAs network, depending on the type of aneurysms and their complications (Figs. **4** and **5**) [57-97]. Moreover, several reports have pointed to the crucial involvement of circRNAs in IAs and TAAs pathogenesis. For example, Qin *et al.* showed the regulatory effect of circ-ARFIP2 on Human Umbilical Arterial Smooth Muscle Cells (HUASMCs) proliferation, migration, and invasion [70]. In particular, in this report, the increased level of circ-ARFIP2 in HUASMCs showed a profound promotion of cell proliferation, migration, and invasion, implying that downregulated circ-ARFIP2 might lead to the reduced migration and proliferation of HUASMCs in IAs. Moreover, it was confirmed that circ-ARFIP2 directly bound to miR-338-3p, and the investigation of miR-338-3p targets in HUASMCs confirmed that Kinase insert Domain Receptor (KDR) was a functionally important target of miR-338-3p. As a result, circ-ARFIP2 was demonstrated to act with the ability to regulate VSMCs function by targeting the miR-338-3p/KDR axis, which provided new insight into the involvement of circ-ARFIP2 in the pathogenesis of IAs. In another study, it was previously reported that the expression level of circ_0021001 and Gremlin1 (GREM1) was upregulated, and the expression level of miR-148b-3p was downregulated in both IAs tissues and HUASMCs [72]. Moreover, by reducing the expression of circ_0021001, the proliferation ability of HUASMCs was suppressed, and apoptosis was induced. Thereby, the inactivation of circ_0021001 could suppress cell growth and induce apoptosis of HUASMCs, partly through modulation of miR-148b-3p/GREM1, presenting circ_0021001 as a promising therapeutic target for IAs. Zhang *et al.* demonstrated that circ_0008285 silencing could suppress angiotensin II (Ang-II)-induced VSMCs apoptosis *via* the miR-150-5p/membrane attached signal protein 1 (BASP1) axis, suggesting a further understanding of the action on the pathogenesis of TAAs [90]. Moreover, the authors demonstrated that extracellular circ_0008285 was secreted by exosomes in VSMCs, which indicates a new exosome-based therapeutic strategy in TAAs.

Macrophages play a crucial role in the resolution of the initial inflammatory phase, which dominates the key pathogenesis of AAAs and includes ECM degeneration and induction of VSMC apoptosis. To clarify the underlying mechanism by which circRNAs promote AAAs formation with macrophages activation, Song *et al.* demonstrated that circCdyl upregulation promoted M1 polarization and stimulated M1-type inflammation by preventing interferon regulatory factor 4 (IRF4) from entering the cell nucleus and acting as a sponge for let-7c to promote CCAAT/enhancer-binding protein delta (C/EBP-δ) expression in macrophages, significantly aggravating Ang-II and calcium chloride (CaCl2)-induced AAAs development [74]. The study by Ma *et al.* delved deeper into the mechanistic aspects of circ_0087352's role in AAAs [86]. The researchers employed various advanced techniques to corroborate their findings. For instance, RNA Immunoprecipitation (RIP) assays were utilized to confirm the interaction between circ_0087352 and miR-149-5p further, ensuring the reliability of the competing endogenous RNA (ceRNA) network model. Subsequent functional assays were conducted to elucidate the impact of circ_0087352 on cellular processes. *in vitro*, experiments demonstrated that silencing circ_0087352 led to a significant reduction in the inflammatory response of THP-1 macrophages, as evidenced by decreased levels of interleukin-6 (IL-6), tumor necrosis factor alpha (TNF-α) and interleukin-1 beta (IL-1β). This underscores the critical role of circ_0087352 in modulating inflammation within the AAAs microenvironment. Moreover, the study explored the downstream effects of nuclear factor-κ B (NF-κB) pathway activation. Chromatin immunoprecipitation (ChIP) assays revealed that the nuclear translocation of NF-κB p65 facilitated by circ_0087352 results in the enhanced transcription of various pro-inflammatory genes. This cascade not only perpetuates inflammation but also contributes to the structural deterioration characteristic of AAAs. The research also extended to *in vivo* models to validate the pathological relevance of circ_0087352. In mouse models of AAAs, overexpression of circ_0087352 recapitulated many of the inflammatory and apoptotic features observed in human specimens. Conversely, knockout models exhibited attenuated disease progression, further solidifying the potential of circ_0087352 as a therapeutic target. Additionally, the clinical applicability of circ_0087352 was assessed. Analysis of patient samples revealed a strong correlation between circ_0087352 expression levels and AAAs severity, suggesting its potential as a diagnostic biomarker. The researchers propose that targeting circ_0087352, either through antisense oligonucleotides or small molecule inhibitors, could provide a novel therapeutic approach for mitigating AAAs progression. In summary, the comprehensive study by Ma *et al.* highlights the multifaceted role of circ_0087352 in AAAs pathogenesis. By acting as a molecular sponge for miR-149-5p, circ_0087352 orchestrates a complex network of inflammatory and apoptotic signals, contributing to disease progression. The findings not only enhance our understanding of AAAs biology but also pave the way for innovative diagnostic and therapeutic strategies centered around circ_0087352.

CircRNAs are a new class of ncRNAs. Recently, studies have focused on detecting changes in circRNAs expression and have identified them as novel players in aneurysms and their complications. Overall, the studies included bioinformatics analyses, rat and mouse models, and human aneurysm tissues. All results showed that the expression of circRNAs was significantly altered in these diseases, and the authors also highlighted their therapeutic utility. However, no changes were found in the expression levels of the same circRNAs or circRNAs-miRNAs-mRNAs pathways in either IAs, TAAs, and AAAs, or in SAH and AAD, implying that further elucidation is needed. Almost all circRNAs realized their functions by combining with miRNAs and target genes. However, due to their potential effects, targeting multiple miRNAs or mRNAs requires further validation for each aneurysm and their effectiveness, especially in humans.

Most of the studies are observational results using bioinformatics analysis demonstrating dysregulation of circRNAs expression in aneurysms and their complications. Experiments exploring the mechanisms of SAH or AAD circRNAs are still partially lacking, and only a few studies have been validated. Thus, it is still unclear whether these changes are causal, prognostic, or simply related to aneurysm formation and their complications. However, we hypothesize that circRNAs may act through the circRNAs-miRNAs-mRNAs axis as novel regulators of the formation and progression of IA, TAA, and AAA and may lead to new treatments in the future.

## 5 CircRNAs AS NON-INVASIVE BIOMARKERS

**Fig. (6):**
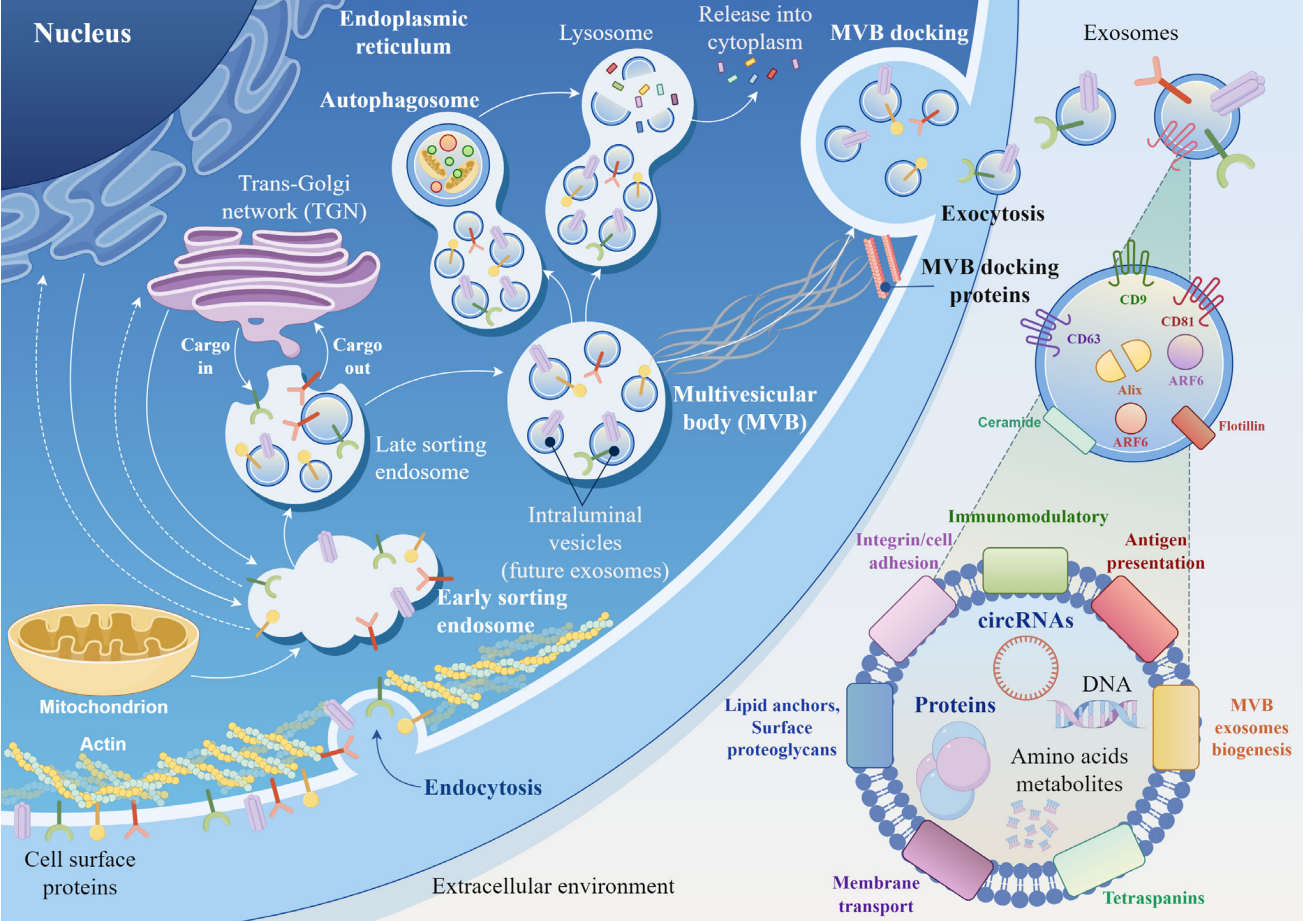
Schematic illustration of active secretion of circular RNAs (circRNAs) from the cell into the extracellular space/fluid within exosomes.

As is already known, circRNAs are not only tissue- and cell-specific, but also exhibit different expression patterns. One circRNA may show high levels of expression in one cell type or one tissue, but it may not be activated or have low levels of expression in another cell or tissue type [98]. This implies in relation to cells or tissues from IAs, TAAs, or AAAs. Moreover, due to their circular chain, unlike linear RNAs, circRNAs are surprisingly stable in the extracellular environment and are found in biological fluids of the human body (Fig. **6**) [99-101]. It has been demonstrated that circulating circRNAs (also called extracellular or cell-free) have many important characteristics of a good biomarker, such as a high degree of sensitivity and specificity to detect early manifestations of pathological conditions, transient changes in expression during the course of the disease, a long half-life in samples, and rapid and cost-effective laboratory detection, for example using real-time polymerase chain reaction (qPCR) [99-101]. Accordingly, they have been shown to be effective non-invasive biomarkers in the diagnosis and prognosis of cardiovascular diseases.

To date, the study of circulating circRNAs as noninvasive biomarkers of IAs and TAAs/AAAs and their complications, such as SAH and dissection, has been demonstrated in several recent studies (Table **3**) [61, 62, 102-105]. Encouraging evidence suggests that deregulation of circRNAs is associated with aneurysm formation and rupture and that these changes in their expression can be observed in human body fluids. It is important to note that altered expression levels of circulating circRNAs in biofluids after SAH may be dynamic over time and maybe most noticeable during the first 3 days after hemorrhage [106]. Clinically, this period is critical in SAH as it is directly related to early acute brain injury, such as Delayed Brain Injury (DBI), which ultimately triggers a cascade of secondary events such as neuroinflammation, oxidative stress, and apoptosis [107].

Current management of aneurysms and their short- and long-term complications as SAH and AAD are based solely on imaging and physical examination. However, this management regimen is highly dependent on the surveillance approach, which may result in treatments ultimately falling outside the optimal window. In addition, imaging and physical examination cannot effectively detect pathological and subtle changes in the vascular wall before serious complications occur. These challenges require new diagnostic tools that can significantly improve the ability to predict, diagnose, prognose, and monitor the clinical course of the disease. There are biomarkers of proteins and various metabolites that include responses to neuronal, angiogenic, coagulation, and inflammatory responses associated with aneurysm formation and its complications. However, the sensitivity and specificity of these biomarkers pose a serious challenge to their effective use in clinical practice [108, 109].

The complexity and heterogeneity of the pathophysiology of IAs, TAAs, or AAAs pose a major challenge in interpreting the functional involvement of circulating circRNAs as non-invasive biomarkers. Unfortunately, the use of circulating circRNAs as non-invasive biomarkers for aneurysms and their complications is currently also limited by the number of studies. In addition, several types of biofluids, including whole blood or plasma/serum and cerebrospinal fluid, can be used to identify biomarkers in aneurysms and their complications. Whole blood or plasma/serum is the preferred source of biomarkers for many different diseases. However, because blood circulates freely within many organ systems, its constituents reflect a more global, systemic condition and may have a high degree of non-specificity. This factor, along with biological fluids, makes it difficult to interpret circulating circRNAs as biomarkers in IAs and SAH. On this side, Cerebrospinal Fluid (CSF) is very specific for the Central Nervous System (CNS) injuries and conditions like IAs and SAH. However, it is more invasive and difficult to analyze compared to blood. Circulating circRNAs in CSF are potentially better candidates as biomarkers for IAs and may be more effective in predicting rupture and functional outcomes [110]. On the other hand, no circulating circRNAs have been identified and studied in the CSF. This is largely due to the heterogeneous nature of IAs and the lack of understanding of the underlying pathophysiological pathways. Future studies will need to consider searching for additional circulating circRNAs or their groups in both CSF and bloodstream to increase the chances of identifying specific biomarkers in IAs.

## CONCLUSION AND FUTURE PERSPECTIVES

**Fig. (7):**
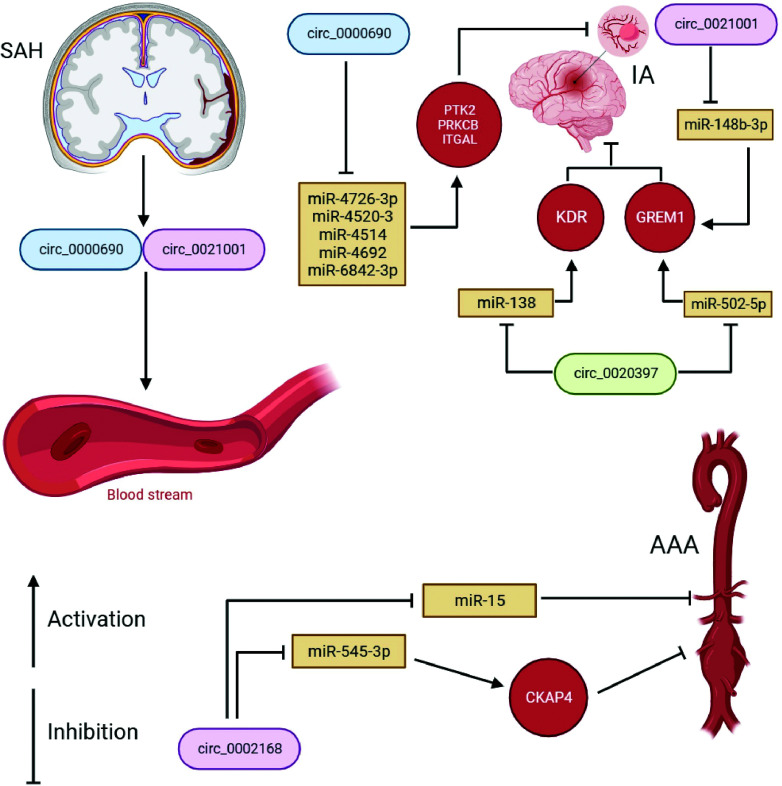
Schematic illustration demonstrating the intersection of circular RNAs (circRNAs) studies in aneurysms. It has been shown that circ_0002168 [68, 73], circ_0020397 [56, 64], circ_0000690 [53, 98] and circ_0021001 [65, 97] have been studied in several studies with the regulation of various signaling pathways in abdominal aortic aneurysm (AAA) and intracranial aneurysm (IA). In addition, circ_0000690 and circ_0021001 were detected in blood as non-invasive biomarkers in subarachnoid hemorrhage (SAH).

It has now become obvious that suppression of the expression of miRNAs and target genes with the participation of circRNAs is an extremely important universal mechanism widely involved in most intracellular signaling pathways. Disturbances of this mechanism are found in a wide variety of human pathologies, including aneurysms. The traditional process of discovering therapeutic agents and diagnostic tools is based on either understanding the molecular or biochemical mechanisms of disease or large-scale profiling and screening. Both approaches have significant challenges in addressing complex, heterogeneous diseases and pathological conditions, including IAs, TAAs, and AAAs. Moreover, altered expression of circRNAs associated with one pathway (circRNAs-miRNAs-mRNAs) may not fully reflect the underlying complexity of the formation and progression of a particular aneurysm. A single circRNA can have hundreds of miRNAs and target genes, and a single miRNA and gene can be the target of multiple circRNAs. However, it should be noted that depending on the type of aneurysm, the same circRNA can play different roles, that is, both a provoking and suppressive role in the formation and progression of a particular aneurysm (Fig. **7**). To understand the biological and pathophysiological mechanisms by which these circRNAs regulate aneurysm formation and progression, it is important to identify and validate additional miRNAs and mRNAs that are aberrantly expressed in aneurysms. Given the tissue-specific expression of circRNAs and possibly other ncRNAs, it is important to consider collecting cell type-specific and aneurysm-specific circRNA samples to determine cell-specific circRNA expression. For example, when possible, collecting circRNAs from ECs, VSMCs, and immune cells would be useful to gain specific insight into cell type-specific changes in expression levels for circRNAs during the development of IAs, TAAs, or AAAs.

In addition, to date, only single circulating circRNAs have been demonstrated as noninvasive biomarkers in the diagnosis and prognosis of IAs, SAH, and AAD. Although there is good evidence that circRNAs are involved in the pathogenesis of aneurysms, the specific mechanisms of their involvement are little known. Modern molecular biological studies should be aimed at identifying targets for individual circRNAs, which will further enable us to understand the fine regulation of signaling pathways, the disturbances of which are associated with a particular aneurysm. These advances will enable us to manipulate the functions of circRNAs for use in diagnostics and therapeutics.

## AUTHORS’ CONTRIBUTIONS

Ilgiz Gareev and Vladimir Chekhonin conceptualized the study, wrote original draft and administered the project. Ozal Beylerli reviewed, edited, investigated, validated, and visualized the study. Aamir Ahmad performed the formal analysis and methodology. Tatiana Ilyasova and Huaizhang Shi contributed to data curation, resources, and acquisition of findings, and Vladimir Chekhonin supervised the study. All authors have read and agreed to the published version of the manuscript.

## Figures and Tables

**Table 1:** Research on the role of circular RNAs (circRNAs) in the formation and progression of intracranial aneurysms (IAs) and subarachnoid hemorrhage (SAH).

**CircRNA**	**Regulation**	**MiRNAs Targets**	**Gene Targets**	**Function**	**References**
circ_000139, circ_101321, circ_072697, circ_069101, and circ_103677	Up	-	mTOR	Involved in the pathogenesis of IA rupture	[58]
circ_0008433 circ_0072309	Up Down	miR-181b-5p and miR-181c-5p miR-519e-5p and miR-516b-5p	MMP2 DDR2 and SAT2	Involved in the pathogenesis of MIA through the VSMCs function control	[59]
circ_0079586 circ_RanGAP1	Up Up	miR-183-5p miR-877-3p	MPO MPO	Involved in the pathogenesis of IA rupture	[60]
circ_0135895 circ_0000682 circ_0000690	Up Down Down	miR-619-3p, miR-4324, miR-5687, miR-3529-5p, and miR-379-5p miR-448, miR-302a-5p, miR-627-3p, and miR-1248 miR-4726-3p, miR-4520-3p, miR-4514, miR-4692, and miR-6842-3p	PTK2, PRKCB, and ITGAL PTK2, PRKCB, and ITGAL PTK2, PRKCB, and ITGAL	Involved in the pathogenesis of MIA through the inflammatory link	[61]
circAFF1	Up	miR-516b	SAV1 and YAP1	Inhibits the proliferation and tube formation and promote apoptosis of ECs. Progression of SAH	[62]
circARF3	Down	miR-31-5p	MyD88 and NF-κB	Neuroprotective effect in the SAH by attenuating BBB destruction and microglia-mediated inflammation	[63]
circ_0020397	Up	miR-138	KDR	Promotes VSMCs proliferation and inhibits cell apoptosis	[64]
circ_0005505	Up	-	MMP2, MMP9, YAP1, and OPN	Promotes the proliferation, migration and suppresses the apoptosis of VSMCs vascular smooth muscle cell. Involved in the pathogenesis of IA rupture	[65]
circ_0031608	Up	-	TNF-α, α-SMA, calponin, and MMP-2	Promotes phenotypic transformation of VSMCs. Important role in the rupture of IA	[66]
circDUS2	Up	-	SMAD/TGF-β/MAPK signaling pathway	Involved in the pathogenesis of IA rupture	[67]
circLIFR	Up	miR-1299	KDR	Enhances VSMCs proliferation, migration, invasion, and impedes apoptosis	[68]
circ_FOXO3	Up	miR-122-5p	KLF6	Suppresses H_2_O_2_-induced proliferation of VSMCs but promotes apoptosis	[69]
circ_DOCK1	Up	miR-409-3p	MCL1	Suppresses H_2_O_2_-induced proliferation of VSMCs and cell apoptosis	[70]
circ_ARFIP2	Up	miR-338-3p	KDR	Promotes in VSMCs proliferation, migration, and invasion	[71]
circ_0020397	Up	miR-502-5p	GREM1	Inhibits VSMC apoptosis	[72]
circ_0021001	Up	miR-148b-3p	GREM1	Inhibits VSMC apoptosis	[73]
circATL1	Down	miR-455	SIRT5	Suppresses of VSMC migration, proliferation, and phenotypic modulation	[74]

**Abbreviations:** ECs, Endothelial cells; HUVECs, Human umbilical vein endothelial cells; HBVSMCs, Human brain vascular smooth muscle cells; PBMCs, Human peripheral blood mononuclear cells**;** HUASMCs, Human umbilical artery smooth muscle cells; MPO, *Myeloperoxidase;* TGF-β, Transforming growth factor beta; MAPK, Mitogen-activated protein kinase; TNF-α, Tumor necrosis factor-alpha; α-SMA, Alpha-smooth muscle actin; MMP-2, Matrix metalloproteinase-2; PTK2, Protein tyrosine kinase 2; PRKCB, Protein kinase Cβ; ITGAL, Integrin subunit αL; SMAD, Mothers against decapentaplegic homolog; MAPK, Mitogen-activated protein kinase; DDR2, Discoidin domain-containing receptor 2; SAT2, Spermidine/spermine N1-acetyltransferase family member 2; KDR, Kinase insert domain receptor; KLF6, Krueppel-like factor 6; MCL1, Myeloid cell leukemia-1; GREM1, Gremlin 1; SIRT5, Sirtuin 5; VSMCs, Vascular smooth muscle cells; MIA, Primary multiple intracranial aneurysm; mTOR, Mammalian target of rapamycin; CSF, Cerebrospinal fluid; MyD88, Myeloid differentiation factor 88; NF-kB, Nuclear factor kappa-light-chain-enhancer of activated B cells; SAH, Subarachnoid hemorrhage; BBB, Blood-brain barrier; HBEC-5i, Human brain-derived endothelial cells; SAV1, Salvador homolog-1; YAP1, Yes-associated protein 1.

**Table 2:** Research on the role of circular RNAs (circRNAs) in the formation and progression of in thoracic aortic aneurysm (TAA), abdominal aortic aneurysm (AAA) and acute aortic dissection (AAD).

**CircRNA**	**Initial Regulation**	**MiRNAs Targets**	**Gene Targets**	**Function**	**References**
circCdyl	Up	let-7c	C/EBP-δ	Accelerates Ang II- and CaCl2-induced AAA formation by promoting M1 polarization and M1-type inflammation	[75]
circ_0005360 circ_0002168	Down Down	miR-181b miR-15	-	Potentially related to the pathogenesis of AAA	[76]
circCCDC66	Down	miR-342-3p	CCDC66	Induces proliferation augmentation and apoptosis reduction of VSMCs	[77]
circRBM33	Up	miR-4268	EPHB2, MMP-2 and TIMP-1	Involves in AAA progression by regulating ECM degradation	[78]
circ_009839, circ_009537, circ_004742, circ_014193, and circ_002856 circ_002813, circ_013683, circ_018331, circ_015216, and circ_001265	Up Down	let-7-2-3p, miR-199b-3p and miR-125a-2-3p let-7-2-3p, miR-199b-3p and miR-125a-2-3p	- -	Potentially related to the pathogenesis of AAA	[79]
circ_0002722 circ_0001837 circ_0000941	Down Down Down	miR-130a-3p and miR-130a b-3p miR-135a-5p and miR-135b-5p miR-135a-5p and miR-135b-5p	PPARG FOXO1 FOXO1	Potentially related to the pathogenesis of AAA with control infiltrating immune cells	[80]
circ_0002168	Down	miR-545-3p	CKAP4	Protective effect on VSMC proliferation. Induces proliferation and suppressed apoptosis in VSMCs of AAA tissue	[81]
circ_0003641	Up	-	ATM and Smooth muscle cell α-actin (α-SMA)	Functions as an “early warning signal” that promotes a switch towards stress resistant VSMCs phenotypes in AAA.	[82]
circ_000595	Up	miR-19a	-	Increase apoptotic rate of VSMCs in AAA	[83]
circChordc1	Down	-	GSK3β/β-catenin pathway	Reduces the proliferation, apoptosis, and phenotypic switching of VSMCs. Inhibits Ang II-induced AAA formation	[84]
circCBFB	Down	miR-28-5p	LYPD3 and GRIA4	Facilitates VSMC proliferation and inhibits VSMC apoptosis in AAA	[85]
circ_0011449 circ_0081968	Up Up	let-7f-5p let-7f-5p	SOD2 and CCR7 PGRMC1, SOD2 and CCR7	Potentially related to the pathogenesis of AAA	[86]
circ_0057691 circ_0092108 circ_0006845 circ_0082182	Up Up Up Up	miR-330-5p miR-330-5p miR-330-5p miR-330-5p	CNN1 CNN1 CNN1 CNN1	Potentially related to the pathogenesis of AAA	[87]
circ_0087352	Up	miR-149-5p	IL-6, CCL2, ERK, and NF-κB	Promotes the inflammatory response of macrophages in AAA	[88]
circ_0090069	Down	miR-1206	RPS27A	Potentially related to the pathogenesis of AAA	[89]
circ-FNDC3B	Up	miR-143-3p	ADAM10	Increases the proliferation, apoptosis, and phenotypic switching of VSMCs. Enhances Ang II-induced AAA formation	[90]
CDR1as	Down	miR-7	CKAP4	Suppresses VSMCs proliferation in AAA	[91]
circ_0008285	Up	miR-150-5p	BASP1	Increases Ang-II-induced VSMCs apoptosis in TAA	[92]
circ_0000595	Up	miR-582-3p	ADAM10	Suppresses VSMC proliferation and promotes VSMCs apoptosis in TAA	[93]
circ_0092291	Down	miR-626	COL4A1	Suppress the Ang II-induced VSMCs dysfunction in AAA	[94]
circRasGEF1B	Up	-	SM22α, ZFP36 and Bcl-2	Novel mechanism underlying macrophages inducing VSMC apoptosis in AAA	[95]
circ_101238	Up	miR-320a	MMP9	Increases the likelihood of developing of TAD	[96]
circPTGR1 circNOX4 circAMN1 circUSP3	Up Up Up Up	- - - -	MMP2 MMP2 MMP2 MMP2	Key roles in the immune cell infiltration (class-switched memory B-cells, macrophages (especially M1 macrophage), and mast cells) in TAD	[97]
сirc_TGFBR2	Down	miR-29a	KLF4	Inhibits VSMCs phenotypic switch and suppresses AAD progression	[98]
circ_0022920	Down	miR-650	TGFβR1	Suppresses the progression of AAD	[99]

**Abbreviations:** VSMCs, Vascular smooth muscle cells; Ang II, Angiotensin II; CaCl2, Calcium chloride; ECM, Extracellular matrix; TAAD, Thoracic aortic dissection; C/EBP-δ, CCAAT/enhancer binding protein delta; CCDC66, Human coiled-coil domain containing 66; EPHB2, EPH Receptor B2; TIMP-1, Tissue inhibitor of metalloproteinase-1; PPARG, Peroxisome proliferator-activated receptor gamma; FOXO1, Forkhead Box O1; CKAP4.

**Table 3:** Circulating circular RNAs (circRNAs) as non-invasive biomarkers for intracranial aneurysms (IAs), thoracic aortic aneurysm (TAA) and abdominal aortic aneurysm (AAA), and their complications.

**Condition**	**CircRNAs**	**Sample**	**Regulation**	**Sensitivity**	**Specificity**	**AUC**	**Important Find**	**References**
TAAD	circPTGR1 circNOX4 circAMN1 circUSP3	Serum	Up Up Up Up	- - - -	- - - -	0.775 0.906 0.875 0.756	Potential biomarker for the diagnosis of TAAD	[97]
IA	circ_0007990	Whole blood	Up	-	-	-	The expression was significantly higher in the unruptured IA with AWE, which also tended to be associated with a larger size of the aneurysm	[104]
IA	circ_0021001	Whole blood	Down	0.81	0.92	0.87	The expression was significantly associated with aneurysm location, aneurysm rupture, Hunt Hess levels, and timing of surgery	[105]
IA	circ_0000690	Whole blood	Down	0.620	0.780	0.752	The expression was significantly associated with GCS, Slicer volume, mFS, Hunt-Hess levels, and surgical type	[106]
SAH	circAFF1	Serum	Up	-	-	-	The expression was higher in patients older than 45 years old and patients with higher Hunt-Hess levels	[107]
SAH	circARF3	Plasma and CSF	Down	-	-	-	The expression was significantly associated with higher Fisher stages	[108]
AAD	circMARK3	Serum	Up	90.0	86.7	0.934	Potential biomarker for the diagnosis of AAD	[109]

**Abbreviations:** AAD, Aortic dissection; SAH, Subarachnoid hemorrhage; CSF, Cerebrospinal fluid; AWE, Aneurysm wall enhancement; GCS, Glasgow Coma Scale; AUC is considered diagnostically significant for the biomarker; /, not mentioned in the article.

## LIST OF ABBREVIATIONS

AAA: Abdominal Aortic Aneurysm
AAD: Aortic Aneurysm Dissection
Ang II: Angiotensin II
AWE: Aneurysm Wall Enhancement
BBB: Blood-Brain Barrier
CaCl_2_: Calcium Chloride
CCDC66: Human Coiled-Coil Domain Containing 66
circular RNAs: circRNAs
CSF: Cerebrospinal Fluid
DDR2: Discoidin Domain-Containing Receptor 2
ECM: Extracellular Matrix
ECs: Endothelial Cells
EPHB2: EPH Receptor B2
FOXO1: Forkhead Box O1
GCS: Glasgow Coma Scale
HUASMCs: Human Umbilical Artery Smooth Muscle Cells
HUVECs: Human Umbilical Vein Endothelial Cells
IA: Intracranial Aneurysm
IAs: Intracranial Aneurysms
KDR: Kinase Insert Domain Receptor
MAPK: Mitogen-Activated Protein Kinase
MCL1: Myeloid Cell Leukemia-1
microRNAs: miRNAs
MMP-2: Matrix Metalloproteinase-2
mTOR: Mammalian Target of Rapamycin
MyD88: Myeloid Differentiation Factor 88
PPARG: Peroxisome Proliferator-Activated Receptor Gamma
PRKCB: Protein Kinase Cβ
PTK2: Protein Tyrosine Kinase 2
SAH: Subarachnoid Hemorrhage
TAA: Thoracic Aortic Aneurysm
TGF-β: Transforming Growth Factor Beta
TIMP-1: Tissue Inhibitor of Metalloproteinase-1
TNF-α: Tumor Necrosis Factor-Alpha
VSMCs: Vascular Smooth Muscle Cells
YAP1: Yes-Associated Protein 1
α-SMA: Alpha-Smooth Muscle Actin
